# Comparison Between Performance Levels for Mathematical Competence: Results for the Sex Variable

**DOI:** 10.3389/fpsyg.2021.663202

**Published:** 2021-06-17

**Authors:** Ramón García Perales, Ascensión Palomares Ruiz

**Affiliations:** Department of Pedagogy, Faculty of Education of Albacete, Castilla-La Mancha University, Albacete, Spain

**Keywords:** math performance, assessment instrument, primary education, educational inclusion, sex

## Abstract

Schools promote all-round education for each of their students. This requires teachers to work on all of the possibilities offered by a subject, including mathematical ability. This process of adjustment and individualization is essential for students who have excellent performance or aptitudes. This study uses an *ex post* facto, descriptive and quantitative research methodology to examine the results of giving the online version of the Evaluation Battery for Mathematical Ability (BECOMA On) to 3795 5th-year primary school students. The sample was selected from 147 Spanish schools from 16 autonomous regions and 2 autonomous municipalities. Three levels of performance were identified, 3 being the highest, and different statistical indices were calculated for each of them. The results were also analyzed according to sex, with statistically significant differences in the highest performance level. In addition, the study highlighted a diagnostic gap in the identification of higher capacity students, a pending challenge for education systems for the educational inclusion of all students.

## Introduction

Educational processes nowadays are characterized by homogeneity and multidimensionality, which makes it difficult to deal with the diverse potentials, needs, and interests in the classroom. Occasionally, there may also be a lack of diagnostics that would allow for the modified, individualized educational responses which are common for students who are highly capable and have high aptitudes (García-Perales and Almeida, 2019). Discovering and working on talent should be a basic objective in an advanced society, and generalizing the detection process and targeting it at the entire school population would be an interesting way of achieving that aim. This study presents an example of that in the field of mathematics. The process allows various situations to be addressed flexibly based on specific student characteristics in order to encourage each student’s cognitive abilities to the highest level.

Mathematics is important because it is applicable in daily life and in solving various types of problems (Cázares et al., 2020), as well as having interdisciplinary connections to other parts of the curriculum (Gilat and Amit, 2013). This generalization to routine everyday contexts is a fundamental aspect of being included as a key skill in education (Méndez et al., 2015). In the case of mathematics, it is included in maths competency and basic competencies in science and technology. Maths competency is defined as “students’ ability to formulate, apply, and interpret mathematics in various contexts. It includes mathematical reasoning and using mathematical concepts, procedures, facts, and tools to describe, explain, and predict various kinds of phenomena” (Ministerio de Educación y Formación Profesional, 2019, p. 17). This definition provides a key aspect of maths evaluation, the measurement of mathematical ability in a broad range of contexts, with a view to highlighting the importance of generalizing what has been learned to a wide variety of situations, familiar or otherwise. The search for constructive, committed, reflective citizenship is a fundamental premise of educational processes, aspects which maths teaching has a strong influence over (Organization for Economic Cooperation, and Development, 2019b). Maths competence has been evaluated in all six editions of the Program for International Student Assessment (PISA) every three years from 2000 to 2018.

The PISA assessments are a fundamental reference for evaluation. The fact that there is a large, worldwide sample for the PISA tests means that the conclusions are extremely important in the development of education policy. Its conceptual framework has been used in many studies (García-Perales, 2014; Ferreira et al., 2017; Rodríguez-Mantilla et al., 2018; Fuentes and Renobell, 2019; Sason et al., 2020). The distinctive characteristics of PISA include (Ministerio de Educación y Formación Profesional, 2019): seeking to guide educational policies, integration of the concept of competence in assessment, the important role of autonomous and lifelong learning, regular deployment, and sensitive international coverage. When interpreting results for each item, PISA uses Item Response Theory (IRT). In this regard, children’s answers are considered according to the child’s level of ability in mathematical competence, in other words, estimates of student performance focus on the type of mathematical tasks that they can solve correctly (Ministerio de Educación y Formación Profesional, 2019). This means performance levels can be identified that allow each child to be placed on a continuous scale of competence for the measured construct (Roderer and Roebers, 2013), showing the percentage of subjects in each level together with their distinctive characteristics, in this case for mathematical competence. This methodology was used with the BECOMA On, the instrument in the present study, in which three performance levels were set based on the scores.

Among the many conclusions from PISA relating to mathematics, reports have stressed that students’ interest in and enjoyment of this area is low, and even noted the presence of personal issues such as anxiety and lack of confidence, especially in girls (Organization for Economic Cooperation, and Development, 2013; Mizala et al., 2015; Organization for Economic Cooperation and Development, 2019d). Throughout the PISA assessments, boys have always had better results than girls in mathematical competence (Ministerio de Educación y Formación Profesional, 2019), with sex being a predictor variable of mathematical performance (Farfán and Simón, 2017; Fuentes and Renobell, 2019; Palomares-Ruiz and García-Perales, 2020). Biological and social factors may act in an interrelated way (Chamorro-Premuzic et al., 2009; Muelas, 2014), including intellectual capacity (Schillinger et al., 2018), complex mathematical reasoning (Desco et al., 2011) and other factors of an individual nature with an impact on the mathematical learning process (Song et al., 2010; Marsh and Martin, 2011; Rodríguez and Guzmán, 2018), school (Carey et al., 2016; Dowker et al., 2016; Schillinger et al., 2018), and family (Pelegrina et al., 2002; Ferreira et al., 2017; Rodríguez-Mantilla et al., 2018). Analyzing students’ mathematical performance according to sex is one objective of the present study.

**FIGURE 1:**
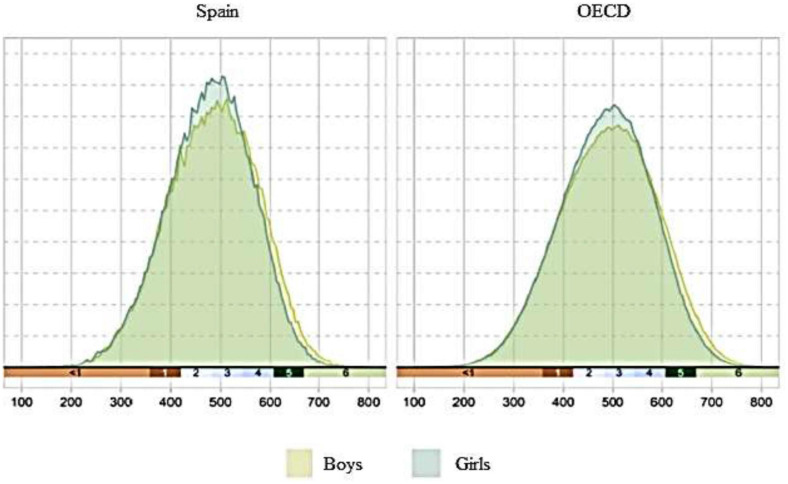
Performance levels and gender in PISA 2018 for Spain and the OECD. Source: Ministerio de Educación y Formación Profesional (2019), p. 89.

The results for mathematics performance in the 2012 PISA tests—the most recent that evaluated mathematics preferentially—and the 2018 tests—the most recent evaluation—are summarized briefly below. In PISA, student results are ranked in seven performance levels: below level 1, 1, 2, 3, 4, 5, and 6. In PISA 2012, Spanish boys scored an average of 492 points and Spanish girls averaged 476 points (Ministerio de Educación, Cultura y Deporte, 2014a). In PISA 2018, Spanish boys averaged 485, while the girls averaged 478. In both cases, the differences between sexes were statistically significant (Ministerio de Educación y Formación Profesional, 2019). Examining these differences more closely, the results for the highest levels 5 and 6 stand out; in PISA 2018, 8% of boys and 5.50% of girls were in one of these two levels. Other studies have also indicated these differences between the sexes in performance and higher ability (Llor et al., 2012; García-Perales and Almeida, 2019; Ministerio de Educación y Formación Profesional, 2020; Palomares-Ruiz and García-Perales, 2020). In contrast, 24.60% of boys and 24.80% of girls were in the lowest levels—1 and below 1—with no statistical significance between the sexes (Ministerio de Educación y Formación Profesional, 2019). As Figure 1 shows, at the higher performance levels the differences between the sexes begin to be more significant, with more boys than girls in those higher levels of mathematics performance (something which is also seen in the OECD average). This is an issue that raises concerns about the potential consequences for future academic and professional choices.

Continuing to look at children with excellent performance in mathematics, in PISA 2012, 8% of Spanish students exhibited excellent performance (6.70% and 1.30% in the two top performing groups), similar figures to previous editions of PISA for mathematics skills, whereas the OECD average was 9.30% and 3.30% in the top two groups (Instituto Nacional de Evaluación Educativa, 2013). In PISA 2018, 6% and 1% of Spanish students were in the top two groups, whereas the OECD mean was 9% and 2% respectively (Ministerio de Educación y Formación Profesional, 2019; Organization for Economic Cooperation, and Development, 2019a,b,c). This raises a fundamental question. In Spain do we not have high performing students? or is our own system not capable of identifying and cultivating them?

What makes a student highly capable at mathematics? PISA 2018, the most recent version, set out the following characteristics for achievement level 6 or higher for maths skills (Ministerio de Educación y Formación Profesional, 2019, p. 64):

“They know how to formulate concepts, generalize and use information based on their research and model complex problems, and they can use their knowledge in relatively atypical contexts. They can simultaneously relate different sources of information and representations, and switch between them flexibly. Students at this level have a high level of mathematical thinking and reasoning. These students can apply this comprehension, as well as their mastery of mathematical operations and symbolic, formal relationships to develop new approaches and strategies to address new situations. Students at this level can consider their actions, and precisely formulate and communicate their actions and thinking about their discoveries, interpretations, arguments, and adaptation to novel situations.”

Other research also influences the conceptualization of the most mathematically capable children (Geary and Brown, 1991; Greenes, 1997; Sriraman, 2003; Rotigel and Fello, 2004; Almeida et al., 2008; Desco et al., 2011; Jaime and Gutiérrez, 2017; Kurnaz, 2018; Ramírez and Cañadas, 2018). Within the field of higher abilities, it is worth paying particular attention to the female population. For example, in Spain, the percentages diagnosed as highly able in school year 2018/19 varied considerably by sex, 65.06% of those identified were boys and 34.94% were girls (Ministerio de Educación y Formación Profesional, 2020). Girls are a higher risk group among the highly able, the identification processes are more detrimental to them (Kerr, 2000; Landau, 2003; Jiménez, 2014) and stereotypes abound (Bian et al., 2017). In addition, even nowadays there are still inequalities in the socialization processes between the sexes (Hadjar et al., 2014; Ministerio de Educación y Formación Profesional, 2019), and girls’ potentials are occasionally undervalued (Pomar et al., 2009). UNESCO (2019, p.72) stated that “the disadvantaging of girls is not based on cognitive ability, but rather on the processes of socialization and learning they grow up with, which shape their identities, beliefs, behaviors, and life choices.”

There is research into maths competency indicating that boys tend to get better results (Preckel et al., 2008; Llor et al., 2012; Instituto Nacional de Evaluación Educativa, 2013; Ministerio de Educación y Formación Profesional, 2019) despite both sexes receiving similar mathematics teaching from the beginning of schooling. Perceptions of and attitudes toward mathematics are particularly important (González-Pienda et al., 2012; Mato et al., 2014; Ministerio de Educación, Cultura y Deporte, 2014b; Preckel et al., 2008; Ministerio de Educación y Formación Profesional, 2019; Cueli et al., 2020; Palomares-Ruiz and García-Perales, 2020), girls can exhibit anxiety and lack confidence in this area (Instituto Nacional de Evaluación Educativa, 2013; Rodríguez-Mantilla et al., 2018). It is essential to consider girls’ levels of attention or execution rates in approaching mathematical tasks (Boaler, 2016; Farfán and Simón, 2017; Hattie et al., 2017; Rodríguez and Guzmán, 2018; Cueli et al., 2020), as well as other motivational and emotional factors (Else-Quest et al., 2010; Rodríguez-Mantilla et al., 2018). Teacher training and practice must consider these discrepancies between aptitude and attitude toward mathematics (Nortes and Nortes, 2013; Rico et al., 2014; Ursini and Ramírez-Mercado, 2017). This variable is a key determiner of educational success in any academic discipline. The more interested students are and the more they believe learning mathematics to be a useful source of knowledge, the better their performance will be (Figueiredo and Guimarães, 2019). This becomes even more important when changing educational stages in the face of deteriorating attitudes toward learning (Mato et al., 2014). Self-efficacy also influences educational development and is a key variable to consider in students’ individual adjustment in the area of mathematics (Ruiz, 2005; Zalazar et al., 2011; Rosário et al., 2012). Better and deeper understanding of these attitudinal and motivational aspects is an essential challenge for mathematics teaching.

Understanding the dimensions that can have an impact on men’s and women’s educational paths is key and affects future academic and professional choices (Hadjar et al., 2014; UNESCO, 2019; García-Perales et al., 2021). It is essential to try to extrapolate from research to answer the question; why is there a difference in the choice of scientific and technical careers between men and women? This study focuses on mathematics, although the same challenge applies to other disciplines such as Science, Technology and Engineering. The goal is to achieve an equal, equitable educational system that allows all students to meet the changing demands of the globalized 21st century society (Ryu et al., 2021), regardless of gender, because there is currently a gender gap in these disciplines (Kijima and Sun, 2020).

The objective of this study was to analyze the results in the BECOMA On from students in the three levels of mathematics achievement. In order to understand and conceptualize these levels of performance, the results were examined in relation to the participants’ sex.

## Materials and Methods

The study used an *ex post* facto, descriptive, quantitative research methodology with the aim of describing the relationships that exist between groups of quantitative data from a series of modulating variables.

### Participants

The study sample was made up of 3795 5th year primary school students, aged around 10-11 years old, from 16 regions in Spain. Each regional education authority selected the schools to participate voluntarily in the study, depending on the schools’ availability to participate and them having suitable technological tools for performing the study. Instruments were applied to class groups in their usual classrooms using online devices. The distribution of the sample by sex was 2002 boys (52.75%) and 1793 girls (47.25%).

The sample was grouped by levels of performance. Based on the results, 3 similarly sized hierarchical levels were set, with 1 being the lowest and 3 being the highest performance. The levels for the BECOMA On are shown in Table 1.

**TABLE 1:** Performance levels in BECOMA On.

Level	Intervals	*n*	%	Valid%	Cumulative%
1	<=30	1319	34.75	34.75	34.75
2	31 – 39	1263	33.28	33.28	68.03
3	40 – 60	1213	31.96	31.96	100.0
	Total	3795	100.0	100.0	

*Source: Authors’ own work.*

The mean level was 1.97 (*SD* = 0.82), with asymmetry of.05, the distribution of the levels followed a symmetric curve, with kurtosis of -1.50, platykurtic distribution with negative excess kurtosis.

### Variables

Mathematics competence was the main variable in this study. It was measured using the BECOMA On. As mentioned above, mathematics has a key role in educational processes, particularly because of its generalization to subjects’ daily lives, a fundamental aspect for effective, autonomous development in society. The other variable used was the participants’ sex, male (M) or female (F).

### Instrument

The BECOMA On is a battery that evaluates mathematical skills in 5th year primary schoolchildren online. It is made up of 30 items spread over 7 evaluation tests: Mathematical interpretation (Items 1-5; Statistics and Probability Dimension), Mental arithmetic (Items 6-11; Arithmetic Dimension), Geometrical properties (Items 12 and 13; Geometry Dimension), Logical numerical series (Items 14-19; Arithmetic Dimension), Discovering algorithms (Items 20 and 21; Arithmetic Dimension), Conventional units (Items 22-27; Magnitudes and Proportionality Dimension), and Logical series of figures (Items 28-30; Geometry Dimension). In establishing the content and evaluation indicators for the items in each dimension, Royal Decree 126/2014, of February 28, which establishes the basic curriculum for Primary Education, was used as a reference (Ministerio de Educación, Cultura y Deporte, 2014c). The instrument is structured as in Table 2.

**TABLE 2:** Instrument structure.

Dimension	Evaluation test	Items	Percentage	Total percentage
Statistics and Probability	1^st^ Mathematical interpretation	1, 2, 3, 4, 5	16.67	16.67
Arithmetic	2^nd^ Mental arithmetic	6, 7, 8, 9, 10, 11	20	46.67
	4^th^ Logical numerical series	14, 15, 16, 17, 18, 19,	20	
	5^th^ Discovering algorithms	20, 21	6.67	
Geometry	3^rd^ Geometrical properties	12, 13	6.67	16.67
	7^th^ Logical series of figures	28, 29, 30	10	
Magnitudes and Proportionality	6^th^ Conventional units	22, 23, 24, 25, 26, 27	20	20
Total	7	30	100	100

*Source: Authors’ own work.*

Each item has a possible score of 0 (wrong), 1 (partially correct), or 2 (correct), giving a possible overall minimum score of 0 and a possible overall maximum score of 60. It takes 41 minutes to do the test. In terms of statistical validity (Palomares-Ruiz and García-Perales, 2020), the instrument had a reliability index of 0.83 using Cronbach’s Alpha, and validity indices between.78 and.86 (content and construct). The Difficulty Index (DI) for each item was as follows:

As Table 3 shows, the battery had a moderate difficulty index (DI = 0.45) and appeared reactive to various levels of difficulty. Item 28 was the most difficult (DI = 0.09) while item 13 was the easiest (DI = 0.75). Item selection was judged by a group of 51 professionals in mathematics from various educational stages, giving an overall validity index for the instrument of 0.81 and a Kappa statistic of 0.82.

**TABLE 3:** Difficulty Index for items in the BECOMA On.

Item	DI
1	0.58
2	0.27
3	0.44
4	0.64
5	0.67
6	0.57
7	0.68
8	0.54
9	0.42
10	0.29
11	0.26
12	0.68
13	0.72
14	0.75
15	0.31
16	0.22
17	0.21
18	0.31
19	0.30
20	0.47
21	0.32
22	0.71
23	0.48
24	0.39
25	0.50
26	0.27
27	0.53
28	0.09
29	0.27
30	0.50
Total	0.45

*Source: Authors’ own work.*

### Procedure

A month before the data collection period, staff at each of the participating schools were given a training course covering the differential characteristics of the battery, and what they had to consider when applying it, with instructions and monitoring times. Data was collected throughout February 2019 through the online application of the instrument.

Consent was obtained from each participating student’s parents or guardians for them to take part in the study, requested on the researchers’ behalf by the director in each school. Subsequently, a list of children with family authorization was kept by the educational administration in each Spanish region.

## Results

Before presenting the results according to the study objectives, the descriptive statistics are presented for each item in the instrument: mean, standard deviation, frequencies and percentages.

**FIGURE 2:**
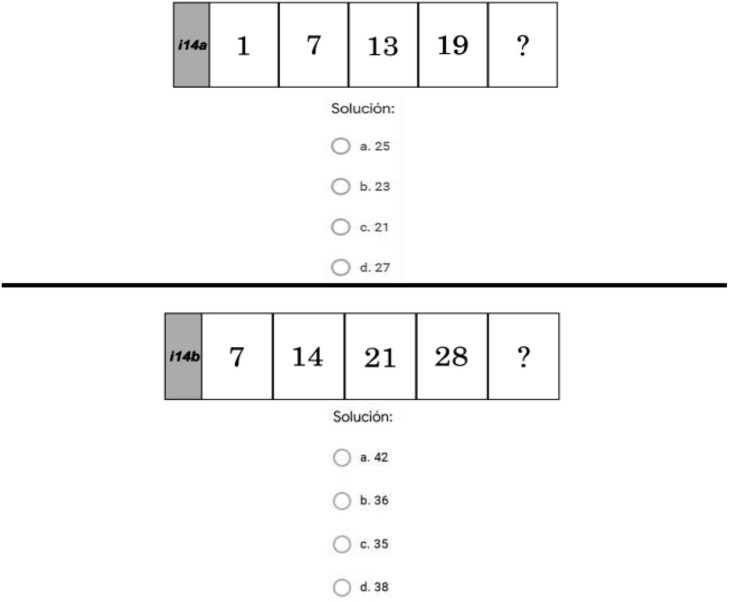
Item 14, the easiest item in the instrument in this study. Authors’ own work (2020).

**FIGURE 3:**
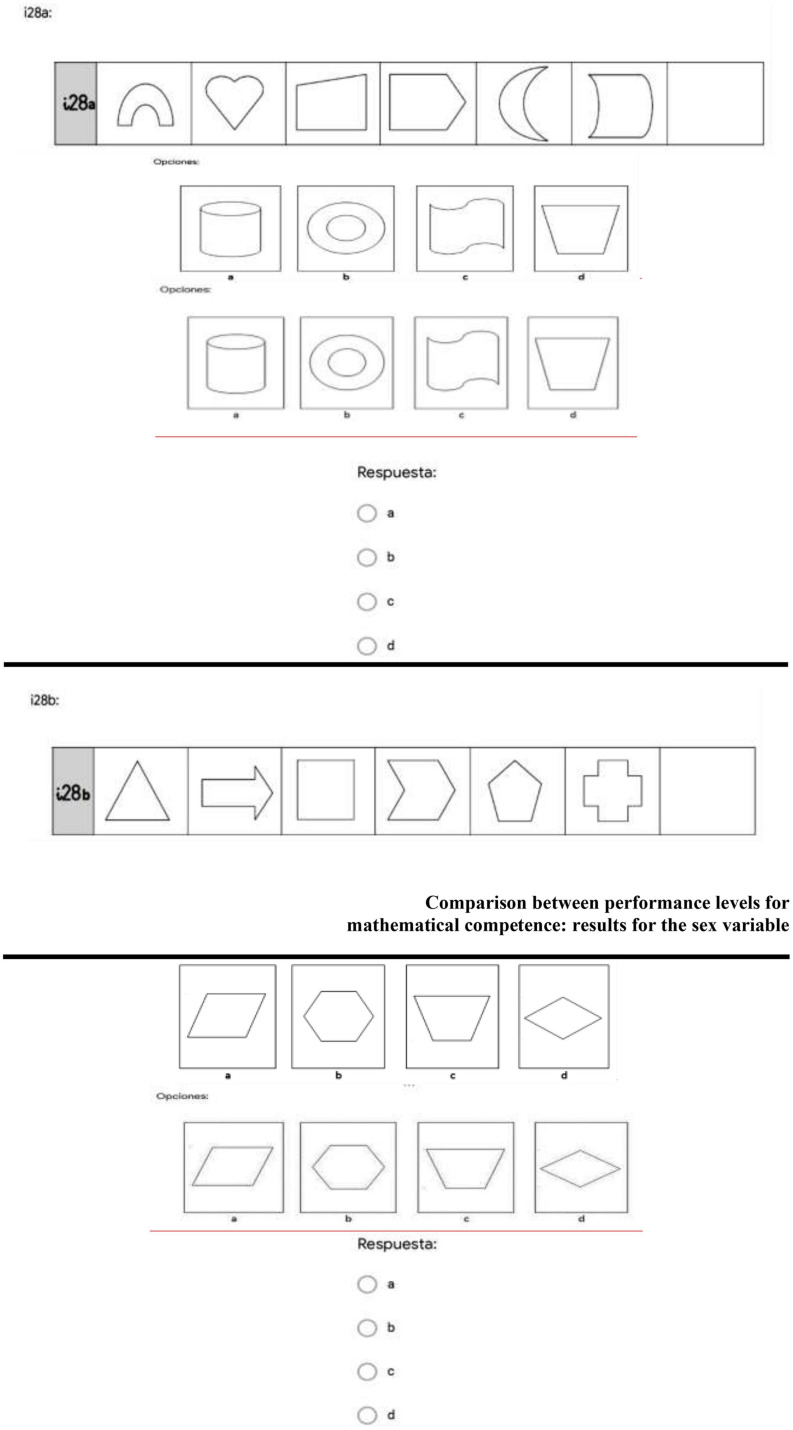
Item 28, the most difficult item in the instrument in this study. Authors’ own work (2020).

As Table 4 indicates, the level of difficulty can be analyzed according to the average results from each item. The easiest items were Items 4 (*M* = 1.49, *SD* = 0.75), 5 (*M* = 1.54, *SD* = 0.71), 7 (*M* = 1.58, *SD* = 0.67), 13 (*M* = 1.61, *SD* = 0.72), and 14 (*M* = 1.63, *SD* = 0.65), and the most difficult items were 11 (*M* = 0.74, *SD* = 0.84), 16 (*M* = 0.81, *SD* = 0.77), 17 (*M* = 0.80, *SD* = 0.76), 21 (*M* = 0.82, *SD* = 0.89), and 28 (*M* = 0.56, *SD* = 0.65). The mean for the battery set was 34.83 (*SD* = 9.69). Figure 2 shows the item with the lowest difficulty level—number 14—and Figure 3 shows the item with the highest difficulty—number 28.

**TABLE 4:** Descriptive statistics of the items of the BECOMA On.

Items			*f*			%			Asim.	Curt.
	*M*	*SD*	0	1	2	0	1	2		
1	1.35	0.84	895	690	2210	23.58	18.18	58.23	–0.72	–1.19
2	0.91	0.78	1344	1438	1013	35.42	37.89	26.69	0.15	–1.36
3	1.14	0.86	1164	954	1677	30.67	25.14	44.19	–0.26	–1.58
4	1.49	0.75	584	767	2444	15.39	20.21	64.40	–1.07	–0.38
5	1.54	0.71	493	775	2527	12.99	20.42	66.59	–1.20	–0.02
6	1.47	0.68	407	1212	2176	10.72	31.94	57.34	–0.90	–0.40
7	1.58	0.67	393	803	2599	10.36	21.16	68.48	–1.33	0.41
8	1.34	0.79	766	967	2062	20.18	25.48	54.33	–0.69	–1.08
9	1.13	0.83	1096	1124	1575	28.88	29.62	41.50	–0.24	–1.51
10	0.93	0.80	1359	1354	1082	35.81	35.68	28.51	0.13	–1.42
11	0.74	0.84	1957	859	979	51.57	22.64	25.80	0.51	–1.39
12	1.43	0.86	955	253	2587	25.16	6.67	68.17	–0.95	–0.99
13	1.61	0.72	525	436	2834	13.83	11.49	74.68	–1.51	0.62
14	1.63	0.65	356	706	2733	9.38	18.60	72.02	–1.50	0.93
15	1.03	0.77	1081	1526	1188	28.48	40.21	31.30	–0.05	–1.32
16	0.81	0.77	1547	1407	841	40.76	37.08	22.16	0.33	–1.25
17	0.80	0.76	1535	1478	782	40.45	38.95	20.61	0.35	–1.18
18	1.06	0.75	955	1647	1193	25.16	43.40	31.44	–0.10	–1.22
19	1.05	0.74	945	1723	1127	24.90	45.40	29.70	–0.08	–1.16
20	1.13	0.89	1271	743	1781	33.49	19.58	46.93	–0.26	–1.68
21	0.82	0.89	1907	655	1233	50.25	17.26	32.49	0.35	–1.65
22	1.48	0.84	862	252	2681	22.71	6.64	70.65	–1.09	–0.69
23	1.16	0.89	1243	716	1836	32.75	18.87	48.38	–0.31	–1.66
24	1.04	0.86	1340	974	1481	35.31	25.67	39.03	–0.07	–1.65
25	1.27	0.81	859	1042	1894	22.64	27.46	49.91	–0.53	–1.27
26	0.98	0.75	1117	1655	1023	29.43	43.61	26.96	0.04	–1.22
27	1.12	0.96	1544	254	1997	40.69	6.69	52.62	–0.24	–1.87
28	0.56	0.65	1996	1474	325	52.60	38.84	8.56	0.73	–0.51
29	0.98	0.75	1103	1657	1035	29.06	43.66	27.27	0.03	–1.22
30	1.27	0.81	883	1020	1892	23.27	26.88	49.86	–0.52	–1.30
Total	34.83	9.69							0.05	–0.39

*Source: Authors’ own work.*

In terms of asymmetry, negative scores predominated—21 of the 30 items—, in other words more values appeared to the left of the mean. In terms of kurtosis, almost all the values—27 of the 30 items and the total score—were negative, a platykurtic distribution with a lower concentration of results around the mean, an interesting aspect when analyzing different levels of performance according to the results.

The results are presented based on the study objectives, first the results in the BECOMA On for the three performance levels and then the descriptive statistics. Following that, each level is examined in relation to sex.

Table 5 shows the results in the BECOMA On for the three performance levels. The frequency and percentages for each performance level are given. There were 1319 students (34.76%) in level 1, 1263 (33.28%) in level 2 and 1213 (31.96%) in level 3.

**TABLE 5:** Frequencies and percentages for the performance levels for each item response.

Item	1						2						3					
	0		1		2		0		1		2		0		1		2	
	*f*	%	*f*	%	*f*	%	*f*	%	*f*	%	*f*	%	*f*	%	*f*	%	*f*	%
1	424	11.17	331	8.72	564	14.86	280	7.38	219	5.77	764	20.13	191	5.03	140	3.69	882	23.24
2	626	16.50	496	13.07	197	5.19	448	11.81	546	14.39	269	7.09	270	7.11	396	10.43	547	14.41
3	612	16.13	348	9.17	359	9.46	381	10.04	340	8.96	542	14.28	171	4.51	266	7.01	776	20.45
4	390	10.28	396	10.43	533	14.04	151	3.98	283	7.46	829	21.84	43	1.13	88	2.32	1082	28.51
5	344	9.06	361	9.51	614	16.18	116	3.06	251	6.61	896	23.61	33	0.87	163	4.30	1017	26.80
6	307	8.09	568	14.97	444	11.70	86	2.27	405	10.67	772	20.34	14	0.37	239	6.30	960	25.30
7	308	8.12	431	11.36	580	15.28	71	1.87	238	6.27	954	25.14	14	0.37	134	3.53	1065	28.06
8	543	14.31	421	11.09	355	9.35	176	4.64	363	9.57	724	19.08	47	1.24	183	4.82	983	25.90
9	704	18.55	441	11.62	174	4.58	321	8.46	427	11.25	515	13.57	71	1.87	256	6.75	886	23.35
10	770	20.29	433	11.41	116	3.06	442	11.65	507	13.36	314	8.27	147	3.87	414	10.91	652	17.18
11	945	24.90	272	7.17	102	2.69	707	18.63	282	7.43	274	7.22	305	8.04	305	8.04	603	15.89
12	532	14.02	148	3.90	639	16.84	290	7.64	71	1.87	902	23.77	133	3.50	34	0.90	1046	27.56
13	291	7.67	224	5.90	804	21.19	163	4.30	150	3.95	950	25.03	71	1.87	62	1.63	1080	28.46
14	289	7.62	400	10.54	630	16.60	61	1.61	216	5.69	986	25.98	6	0.16	90	2.37	1117	29.43
15	717	18.89	484	12.75	118	3.11	303	7.98	623	16.42	337	8.88	61	1.61	419	11.04	733	19.31
16	812	21.40	424	11.17	83	2.19	547	14.41	512	13.49	204	5.38	188	4.95	471	12.41	554	14.60
17	794	20.92	460	12.12	65	1.71	522	13.75	563	14.84	178	4.69	219	5.77	455	11.99	539	14.20
18	638	16.81	542	14.28	139	3.66	259	6.82	638	16.81	366	9.64	58	1.53	467	12.31	688	18.13
19	631	16.63	563	14.84	125	3.29	250	6.59	674	17.76	339	8.93	64	1.69	486	12.81	663	17.47
20	574	15.13	318	8.38	427	11.25	458	12.07	259	6.82	546	14.39	239	6.30	166	4.37	808	21.29
21	820	21.61	265	6.98	234	6.17	694	18.29	219	5.77	350	9.22	393	10.36	171	4.51	649	17.10
22	536	14.12	146	3.85	637	16.79	254	6.69	85	2.24	924	24.35	72	1.90	21	0.55	1120	29.51
23	562	14.81	283	7.46	474	12.49	422	11.12	251	6.61	590	15.55	259	6.82	182	4.80	772	20.34
24	653	17.21	342	9.01	324	8.54	459	12.09	345	9.09	459	12.09	228	6.01	287	7.56	698	18.39
25	495	13.04	430	11.33	394	10.38	278	7.33	364	9.59	621	16.36	86	2.27	248	6.53	879	23.16
26	580	15.28	516	13.60	223	5.88	370	9.75	604	15.92	289	7.62	167	4.40	535	14.10	511	13.47
27	767	20.21	143	3.77	409	10.78	523	13.78	78	2.06	662	17.44	254	6.69	33	0.87	926	24.40
28	836	22.03	428	11.28	55	1.45	666	17.55	504	13.28	93	2.45	494	13.02	542	14.28	177	4.66
29	572	15.07	608	16.02	139	3.66	383	10.09	578	15.23	302	7.96	148	3.90	471	12.41	594	15.65
30	517	13.62	443	11.67	359	9.46	267	7.04	368	9.70	628	16.55	99	2.61	209	5.51	905	23.85

*Source: Authors’ own work.*

Table 5 gives the totals for each level and response option. In the mean results for all items, 15.45% of the students were in level 1 and scored 0, 10.25% were in level 1 and scored 1, and 9.06% were in level 1 and scored 2; 9.09% of students were in level 2 and scored 0, 9.63% were in level 2 and scored 1, and 14.56% were in level 2 and scored 2; 3.99% of the students were in level 3 and scored 0, 6.97% were in level 3 and scored 1, and 21.01% were in level 3 and scored 2. Level 1 and 2 student responses were more erratic and reflected a clear difference between levels. To determine statistically significant differences, Table 6 shows the means, standard deviations and the results of the ANOVA test.

**TABLE 6:** ANOVA Test comparing performance levels.

Item	1		2		3		*F*	*df*	*p*	Eta^2^	Direction
	*M*	*SD*	*M*	*SD*	*M*	*SD*					
1	1.11	0.86	1.38	0.82	1.57	0.75	104.44	3794	0.000***	0.05	1 < 2 < 3
2	0.67	0.72	0.86	0.74	1.23	0.79	177.49	3794	0.000***	0.09	1 < 2 < 3
3	0.81	0.84	1.13	0.85	1.50	0.73	231.38	3794	0.000***	0.11	1 < 2 < 3
4	1.11	0.83	1.54	0.70	1.86	0.44	385.76	3794	0.000***	0.17	1 < 2 < 3
5	1.20	0.83	1.62	0.65	1.81	0.46	275.77	3794	0.000***	0.13	1 < 2 < 3
6	1.10	0.75	1.54	0.62	1.78	0.44	390.02	3794	0.000***	0.17	1 < 2 < 3
7	1.21	0.79	1.70	0.57	1.87	0.37	406.32	3794	0.000***	0.18	1 < 2 < 3
8	0.86	0.81	1.43	0.72	1.77	0.50	560.44	3794	0.000***	0.23	1 < 2 < 3
9	0.60	0.71	1.15	0.80	1.67	0.58	735.85	3794	0.000***	0.28	1 < 2 < 3
10	0.50	0.65	0.90	0.77	1.42	0.70	527.96	3794	0.000***	0.22	1 < 2 < 3
11	0.36	0.62	0.66	0.81	1.25	0.83	443.07	3794	0.000***	0.19	1 < 2 < 3
12	1.08	0.94	1.48	0.84	1.75	0.64	216.11	3794	0.000***	0.10	1 < 2 < 3
13	1.39	0.82	1.62	0.70	1.83	0.51	128.86	3794	0.000***	0.06	1 < 2 < 3
14	1.26	0.79	1.73	0.54	1.92	0.29	427.39	3794	0.000***	0.18	1 < 2 < 3
15	0.55	0.65	1.03	0.71	1.55	0.59	749.88	3794	0.000***	0.28	1 < 2 < 3
16	0.45	0.61	0.73	0.72	1.30	0.72	505.69	3794	0.000***	0.21	1 < 2 < 3
17	0.45	0.59	0.73	0.69	1.26	0.74	471.40	3794	0.000***	0.20	1 < 2 < 3
18	0.62	0.67	1.08	0.70	1.52	0.59	595.95	3794	0.000***	0.24	1 < 2 < 3
19	0.62	0.65	1.07	0.68	1.49	0.60	586.50	3794	0.000***	0.24	1 < 2 < 3
20	0.89	0.86	1.07	0.89	1.47	0.80	151.60	3794	0.000***	0.07	1 < 2 < 3
21	0.56	0.78	0.73	0.87	1.21	0.90	200.13	3794	0.000***	0.09	1 < 2 < 3
22	1.08	0.94	1.53	0.81	1.86	0.49	330.72	3794	0.000***	0.15	1 < 2 < 3
23	0.93	0.88	1.13	0.89	1.42	0.82	102.04	3794	0.000***	0.05	1 < 2 < 3
24	0.75	0.82	1.00	0.85	1.39	0.78	192.01	3794	0.000***	0.09	1 < 2 < 3
25	0.92	0.82	1.27	0.80	1.65	0.61	299.49	3794	0.000***	0.14	1 < 2 < 3
26	0.73	0.73	0.94	0.72	1.28	0.69	192.52	3794	0.000***	0.09	1 < 2 < 3
27	0.73	0.90	1.11	0.96	1.55	0.82	267.18	3794	0.000***	0.12	1 < 2 < 3
28	0.41	0.57	0.55	0.63	0.74	0.70	86.91	3794	0.000***	0.04	1 < 2 < 3
29	0.67	0.66	0.94	0.73	1.37	0.69	321.88	3794	0.000***	0.14	1 < 2 < 3
30	0.88	0.81	1.29	0.79	1.66	0.62	348.70	3794	0.000***	0.15	1 < 2 < 3
Total	24.49	4.67	34.93	2.58	45.97	4.76	8553.78	3794	0.000***	0.82	1 < 2 < 3

* Significant at 5% (*p* < 0.05).

**Significant at 1% (*p* < 0.01), and

***Significant at 0.01% (*p* < 0.001).

*Source: Authors’ own work (2020).*

The students in level 1 had a mean score of 24.49 (*SD* = 4.67), in level 2 the mean score was 34.93 (*SD* = 2.58), and in level 3 it was 45.97 (*SD* = 4.76). The students in level 3 had higher mean scores in all items. Furthermore, the differences between levels were statistically significant in all items, *p* < 0.001, with the level 3 students scoring higher. To complete the characterization of these three groups of students, another variable, students’ sex, was used for comparison between levels.

The sex distribution of the original sample of 3795 students was 2002 boys (52.75%) and 1793 girls (47.25%). The mean score in the instrument for boys was 35.18 (*SD* = 10.08) and for girls it was 34.44 (*SD* = 9.22), with a *p*-Value < 0.05. To more closely examine the significance of the differences between the sexes, the results were analyzed according to each level of performance. The frequencies and percentages for each sex in each of the three levels are given below.

At level 1 performance, there was little difference in the proportions for each score (Table 7): 23.12% of the responses were boys scoring 0, 15.68% were boys scoring 1, and 13.21% were boys scoring 2; 21.33% of the responses were girls scoring 0, 13.80% were girls scoring 1, and 12.86% were girls scoring 2. Looking at the frequencies of scores of 2 for both sexes, there were differences. There were more boys scoring 2 in items 15 (boys 5.69% and girls 3.26%), 20 (boys 17.44% and girls 14.94%), 22 (boys 25.78% and girls 22.52%), and 27 (boys 17.66% and girls 13.34%). More girls scored 2 in items 1 (boys 20.55% and girls 22.21%), 7 (boys 20.70% and girls 23.28%), 8 (boys 12.66% and girls 14.25%), and 12 (boys 23.43% and girls 25.02%).

**TABLE 7:** Frequencies and percentages by sex from students with level 1 performance.

Item	Boys						Girls					
	0		1		2		0		1		2	
	*f*	%	*f*	%	*f*	%	*f*	%	*f*	%	*f*	%
1	234	17.74	181	13.72	271	20.55	190	14.40	150	11.37	293	22.21
2	331	25.09	261	19.79	94	7.13	295	22.37	235	17.82	103	7.81
3	334	25.32	169	12.81	183	13.87	278	21.08	179	13.57	176	13.34
4	195	14.78	219	16.60	272	20.62	195	14.78	177	13.42	261	19.79
5	178	13.50	196	14.86	312	23.65	166	12.59	165	12.51	302	22.90
6	167	12.66	293	22.21	226	17.13	140	10.61	275	20.85	218	16.53
7	170	12.89	243	18.42	273	20.70	138	10.46	188	14.25	307	23.28
8	301	22.82	218	16.53	167	12.66	242	18.35	203	15.39	188	14.25
9	359	27.22	228	17.29	99	7.51	345	26.16	213	16.15	75	5.69
10	391	29.64	241	18.27	54	4.09	379	28.73	192	14.56	62	4.70
11	491	37.23	147	11.14	48	3.64	454	34.42	125	9.48	54	4.09
12	291	22.06	86	6.52	309	23.43	241	18.27	62	4.70	330	25.02
13	155	11.75	127	9.63	404	30.63	136	10.31	97	7.35	400	30.33
14	161	12.21	217	16.45	308	23.35	128	9.70	183	13.87	322	24.41
15	365	27.67	246	18.65	75	5.69	352	26.69	238	18.04	43	3.26
16	403	30.55	233	17.66	50	3.79	409	31.01	191	14.48	33	2.50
17	428	32.45	219	16.60	39	2.96	366	27.75	241	18.27	26	1.97
18	315	23.88	293	22.21	78	5.91	323	24.49	249	18.88	61	4.62
19	321	24.34	291	22.06	74	5.61	310	23.50	272	20.62	51	3.87
20	284	21.53	172	13.04	230	17.44	290	21.99	146	11.07	197	14.94
21	428	32.45	142	10.77	116	8.79	392	29.72	123	9.33	118	8.95
22	262	19.86	84	6.37	340	25.78	274	20.77	62	4.70	297	22.52
23	298	22.59	152	11.52	236	17.89	264	20.02	131	9.93	238	18.04
24	349	26.46	171	12.96	166	12.59	304	23.05	171	12.96	158	11.98
25	267	20.24	225	17.06	194	14.71	228	17.29	205	15.54	200	15.16
26	294	22.29	279	21.15	113	8.57	286	21.68	237	17.97	110	8.34
27	376	28.51	77	5.84	233	17.66	391	29.64	66	5.00	176	13.34
28	432	32.75	227	17.21	27	2.05	404	30.63	201	15.24	28	2.12
29	291	22.06	330	25.02	65	4.93	281	21.30	278	21.08	74	5.61
30	278	21.08	236	17.89	172	13.04	239	18.12	207	15.69	187	14.18

*Source: Authors’ own work.*

For the level 2 students, the frequencies and percentages for each response option were as follows (Table 8):

**TABLE 8:** Frequencies and percentages by sex from students with level 2 performance.

Item	Boys						Girls					
	0		1		2		0		1		2	
	*f*	%	*f*	%	*f*	%	*f*	%	*f*	%	*f*	%
1	137	10.85	104	8.23	395	31.27	143	11.32	115	9.11	369	29.22
2	224	17.74	279	22.09	133	10.53	224	17.74	267	21.14	136	10.77
3	206	16.31	161	12.75	269	21.30	175	13.86	179	14.17	273	21.62
4	82	6.49	156	12.35	398	31.51	69	5.46	127	10.06	431	34.13
5	80	6.33	130	10.29	426	33.73	36	2.85	121	9.58	470	37.21
6	37	2.93	224	17.74	375	29.69	49	3.88	181	14.33	397	31.43
7	45	3.56	139	11.01	452	35.79	26	2.06	99	7.84	502	39.75
8	103	8.16	196	15.52	337	26.68	73	5.78	167	13.22	387	30.64
9	151	11.96	214	16.94	271	21.46	170	13.46	213	16.86	244	19.32
10	221	17.50	256	20.27	159	12.59	221	17.50	251	19.87	155	12.27
11	364	28.82	153	12.11	119	9.42	343	27.16	129	10.21	155	12.27
12	160	12.67	41	3.25	435	34.44	130	10.29	30	2.38	467	36.98
13	89	7.05	89	7.05	458	36.26	74	5.86	61	4.83	492	38.95
14	33	2.61	108	8.55	495	39.19	28	2.22	108	8.55	491	38.88
15	129	10.21	293	23.20	214	16.94	174	13.78	330	26.13	123	9.74
16	240	19.00	279	22.09	117	9.26	307	24.31	233	18.45	87	6.89
17	242	19.16	284	22.49	110	8.71	280	22.17	279	22.09	68	5.38
18	102	8.08	328	25.97	206	16.31	157	12.43	310	24.54	160	12.67
19	109	8.63	337	26.68	190	15.04	141	11.16	337	26.68	149	11.80
20	219	17.34	147	11.64	270	21.38	239	18.92	112	8.87	276	21.85
21	340	26.92	122	9.66	174	13.78	354	28.03	97	7.68	176	13.94
22	112	8.87	53	4.20	471	37.29	142	11.24	32	2.53	453	35.87
23	241	19.08	129	10.21	266	21.06	181	14.33	122	9.66	324	25.65
24	223	17.66	170	13.46	243	19.24	236	18.69	175	13.86	216	17.10
25	116	9.18	191	15.12	329	26.05	162	12.83	173	13.70	292	23.12
26	172	13.62	318	25.18	146	11.56	198	15.68	286	22.64	143	11.32
27	246	19.48	41	3.25	349	27.63	277	21.93	37	2.93	313	24.78
28	351	27.79	231	18.29	54	4.28	315	24.94	273	21.62	39	3.09
29	203	16.07	299	23.67	134	10.61	180	14.25	279	22.09	168	13.30
30	150	11.88	204	16.15	282	22.33	117	9.26	164	12.98	346	27.40

*Source: Authors’ own work.*

At level 2 the results were similar to level 1 in terms of sex, with small differences between boys and girls. 13.53% of responses were boys scoring 0, 14.98% were boys scoring 1, and 21.84% were boys scoring 2; 13.78% were girls scoring 0, 13.95% were girls scoring 1, and 21.91% were girls scoring 3. Looking at the frequencies of scores of 2 for each item, there were also differences between the sexes. More boys scored 2 in items 15 (boys 16.94% and girls 9.74%), 17 (boys 8.71% and girls 5.38%), 18 (boys 16.31% and girls 12.67%), and 19 (boys 15.04% and girls 11.80%). More girls scored 2 in items 7 (boys 35.79% and girls 39.75%), 8 (boys 26.68% and girls 30.64%), 23 (boys 21.06% and girls 25.65%), and 30 (boys 22.33% and girls 27.40%).

For the level 3 students, the frequencies and percentages for each response option were as follows (Table 9):

**TABLE 9:** Frequencies and percentages by sex from students with level 3 performance.

Item	Boys						Girls					
	0		1		2		0		1		2	
	*f*	%	*f*	%	*f*	%	*f*	%	*f*	%	*f*	%
1	127	10.47	71	5.85	482	39.74	64	5.28	69	5.69	400	32.98
2	155	12.78	213	17.56	312	25.72	115	9.48	183	15.09	235	19.37
3	100	8.24	162	13.36	418	34.46	71	5.85	104	8.57	358	29.51
4	24	1.98	46	3.79	610	50.29	19	1.57	42	3.46	472	38.91
5	15	1.24	89	7.34	576	47.49	18	1.48	74	6.10	441	36.36
6	7	0.58	143	11.79	530	43.69	7	0.58	96	7.91	430	35.45
7	8	0.66	86	7.09	586	48.31	6	0.49	48	3.96	479	39.49
8	28	2.31	106	8.74	546	45.01	19	1.57	77	6.35	437	36.03
9	43	3.54	140	11.54	497	40.97	28	2.31	116	9.56	389	32.07
10	89	7.34	223	18.38	368	30.34	58	4.78	191	15.75	284	23.41
11	166	13.69	165	13.60	349	28.77	139	11.46	140	11.54	254	20.94
12	76	6.27	25	2.06	579	47.73	57	4.70	9	0.74	467	38.50
13	46	3.79	38	3.13	596	49.13	25	2.06	24	1.98	484	39.90
14	2	0.16	49	4.04	629	51.85	4	0.33	41	3.38	488	40.23
15	20	1.65	180	14.84	480	39.57	41	3.38	239	19.70	253	20.86
16	77	6.35	253	20.86	350	28.85	111	9.15	218	17.97	204	16.82
17	99	8.16	237	19.54	344	28.36	120	9.89	218	17.97	195	16.08
18	25	2.06	243	20.03	412	33.97	33	2.72	224	18.47	276	22.75
19	25	2.06	242	19.95	413	34.05	39	3.22	244	20.12	250	20.61
20	126	10.39	90	7.42	464	38.25	113	9.32	76	6.27	344	28.36
21	209	17.23	91	7.50	380	31.33	184	15.17	80	6.60	269	22.18
22	25	2.06	12	0.99	643	53.01	47	3.87	9	0.74	477	39.32
23	156	12.86	104	8.57	420	34.62	103	8.49	78	6.43	352	29.02
24	128	10.55	147	12.12	405	33.39	100	8.24	140	11.54	293	24.15
25	39	3.22	134	11.05	507	41.80	47	3.87	114	9.40	372	30.67
26	81	6.68	308	25.39	291	23.99	86	7.09	227	18.71	220	18.14
27	124	10.22	17	1.40	539	44.44	130	10.72	16	1.32	387	31.90
28	289	23.83	298	24.57	93	7.67	205	16.90	244	20.12	84	6.92
29	94	7.75	277	22.84	309	25.47	54	4.45	194	15.99	285	23.50
30	66	5.44	138	11.38	476	39.24	33	2.72	71	5.85	429	35.37

*Source: Authors’ own work.*

At level 3 there were greater differences between the sexes, with boys scoring higher than girls. 6.78% of responses were boys scoring 0, 11.89% were boys scoring 1, and 37.38% were boys scoring 2; 5.70% of responses were girls scoring 0, 9.91% were girls scoring 1, and 28.33% were girls scoring 2. Looking at the scores of 2 for each item, there were large differences between the sexes. This was notable in items 15 (boys 39.57% and girls 20.86%), 19 (boys 34.05% and girls 20.61%), 22 (boys 53.01% and girls 39.32%) and 27 (boys 44.44% and girls 31.90%). At this level, the differences were smaller in items 3 (boys 34.46% and girls 29.51%), 26 (boys 23.99% and girls 18.14%), 28 (boys 7.67% and girls 6.92%), 29 (boys 25.47% and girls 23.50%) and 30 (boys 39.24% and girls 35.37%). There were no items in which more girls scored 2 than boys.

Once the frequencies were established for each level by sex, a *t*-test was performed to determine whether there were statistically significant differences according to sex. The results are given in Table 10.

**TABLE 10:** *t*-Test by sex between performance levels 1, 2, and 3.

Sex	1		2		3		*t*	*df*	*p*
	*f*	%	*f*	%	*f*	%			
Male	686	18.08	636	16.76	680	17.92	-1.99	3793	0.040*
Female	633	16.68	627	16.52	533	14.04			

*Significant at 5% (*p* < 0.05).

**Significant at 1% (*p* < 0.01), and

***Significant at 0.01% (*p* < 0.001).

*Source: Authors’ own work (2020).*

As Table 10 shows, there were statistically significant differences at *p* < 0.05. This significance was due to an unequal frequency between the sexes at performance level 3, where there were 680 boys (17.92%) and 533 girls (14.04%). In the other two levels, 1 and 2, the results were more similar, level 1 included 686 (18.08%) boys and 633 (16.68%) girls, while level 2 included 636 boys (16.76%) and 627 girls (16.52%). According to statistics from the Ministry for Education (Ministerio de Educación y Formación Profesional, 2020) for non-university education in school year 2018/19 (the most recent available data), of the 35494 students identified as highly capable, 23092 were boys (65.06%), and 12402 were girls (34.94%). This reflects a continuing disparity between the sexes in the identification of highly capable students, with the diagnostic process being detrimental to girls. This indicates an inequality in education and the need to examine causal factors more deeply.

## Discussion

Society’s scientific and technological progress requires highly qualified professionals (Frey and Osborne, 2017) as there are constant innovations and shifting requirements (Macià and Garreta, 2018). Schools are fundamental in developing students’ talents (Mandelman et al., 2010). The most recent Spanish education laws address educational needs, and addressing and adapting to students’ needs from the very beginnings of schooling is fundamental. Equality and innovation promote quality and social development (Organization for Economic Cooperation, and Development, 2019a), and evaluation and research help monitor them in order to establish educational policies (Schleicher, 2018; Harju-Luukkainen et al., 2020).

This study focused on the analysis of mathematics skills in the three groups of students identified by performance following the application of the BECOMA On, an instrument with high indices of reliability and validity. Understanding the potential of the students in these three levels is of significant social and educational interest and understanding the complexity of the mathematical approaches and strategies they use in problem solving is fundamental (Jaime and Gutiérrez, 2017). Initially, the results were as expected, students in the highest level—3—demonstrated better results and assessments than students in levels 1 and 2. What is interesting is the presence of various statistically significant differences.

Just over a third, 1319 students (34.76%), were identified as belonging to performance level 1, 1263 (33.28%) to level 2 and 1213 (31.96%) to level 3. Comparing the results of these three groups, statistically significant differences were found, *p* < 0.001; level 3 students had higher scores in all of the items in the instrument. Level 3 students were the most capable in mathematics. It is important to consider the processes for identifying these highly capable students. From time to time, unfortunately, their potentials, needs, and interests seem to be neglected in the learning and teaching process, and occasionally there are various serious adaptation problems (Pomar et al., 2009; García-Perales and Almeida, 2019).

To complete the characterization of these three groups of students, the levels were compared in relation to students’ sex. In the study, 52.75% of the participating sample were boys and 47.25% girls. In performance levels 1 and 2, there were few discrepancies in performance between the sexes, with varying differences in favor of one sex or the other. However, at level 3, there were greater differences, and it was the boys who had the highest scores in all items. Boys in level 3 had 6.78% of responses scoring 0, 11.89% scoring 1, and 37.38% scoring 2. For the girls in level 3, the percentages were 5.70%, 9.91% and 28.33% for scores of 0, 1, and 2 respectively. This resulted in statistically significant differences, *p* < 0.05, since at performance level 3 or higher, there were 680 boys (17.92%) compared to 533 girls (14.04%). This reflects a continuing disparity between the sexes in the higher achievement levels for Mathematics, also seen in other research (Baye and Monseur, 2016; Hyde, 2016; Ministerio de Educación y Formación Profesional, 2019), demonstrating an inequality in education and the need to examine causal factors in depth (Calvo, 2018).

In short, the instrument used is functional and original because it establishes a relationship between assessment and mathematical and digital skills. Its close connection with the Spanish school curriculum for the 5th grade of primary education gives it a valuable practical component for use in developing educational practices. The detection of learning needs and potentials, in this case for mathematics using online evaluation, is key because of mathematics’ instrumental and interdisciplinary nature, and it opens up an interesting path for the generalization and application of such instruments.

## Conclusion

Schools must develop educational practices that allow inclusive, quality education for all (Franco et al., 2017; Arnaiz-Sánchez et al., 2018, 2020). Educational administrations must ensure all students achieve functional and meaningful learning, making it a priority to support the existence of equitable, democratic schooling adjusted to each student’s needs and characteristics. Educational policies must be directed toward achieving this end. In this regard, it is essential to consider all the variables that influence the teaching and learning processes, including student sex (Hadjar et al., 2014; Farfán and Simón, 2017; Palomares-Ruiz and García-Perales, 2020), with a view to rethinking actions to foster improvement in academic performance and to promote innovation in education.

As noted in the introduction, biological factors, such as intelligence or certain personality traits, and contextual factors, such as stereotypes and the family itself, may explain differences between the sexes in mathematical performance, especially at higher performance levels. In this regard, analyzing the contexts in which boys and girls socialize is fundamental for studying these differences between the sexes (Hadjar et al., 2014; Mizala et al., 2015; Palomares-Ruiz and García-Perales, 2020), an issue that should be approached from various perspectives (Del Río et al., 2016). In addition, the differences between the sexes highlight the need to rethink educational practices from the perspective of equality and innovation, trying to prevent mathematical learning from leading to academic and professional segregation (Cantoral et al., 2014). In this regard, working on STEAM skills (Science, Technology, Engineering, Arts and Mathematics) may be a useful approach for promoting coeducation and gender equality in education (UNESCO, 2019; Ryu et al., 2021), including non-formal education (Juvera and Hernández-López, 2021), and may be generalizable to highly capable mathematics students (García-Perales and Almeida, 2019). Teacher training in teaching mathematics is especially important (Monroy and Marroquín, 2020) and is a key aspect for teaching and learning in the other STEAM fields (Román-Graván et al., 2020; Hernández-Barco et al., 2021; Ortiz-Revilla and Greca, 2021), in which women are underrepresented (Lehman et al., 2017; Botella et al., 2019; McCullough, 2020).

The large sample participating in this study underlines the importance of using ability tests for diagnostic processes, in this case for Mathematics. The generalization of specific activities for any schoolchild, whatever their abilities, means starting a process of educational adaptation and individualization (Díez and Jiménez, 2018; Torres, 2018). Currently, educational processes are characterized by their complexity and multidimensionality, with multiple factors that can have an impact on teaching and learning as part of mathematics teaching (Palomares-Ruiz and García-Perales, 2020). For this reason, it would be advisable to expand the variables of analysis in future studies with BECOMA On, and include variables such as academic performance, teachers’ and students’ perceptions of students’ interest in and motivation for mathematics, and whether highly capable students are detected. In addition, future studies will seek to generalize the application of this instrument to other educational levels, the sex of the students will be a fundamental variable. Generalizing studies for this variable to other educational levels would add weight to the results from this study. In addition, attempts will be made to perform repeated-measure replication study designs, similar to those used in other studies, using the written version of this instrument (García-Perales et al., 2020, 2021). Identifying any student’s potential for mathematics helps to offer an individualized educational response, which is a priority of inclusive, high-quality education.

## Data Availability Statement

The original contributions presented in the study are included in the article/supplementary material, further inquiries can be directed to the corresponding author/s.

## Ethics Statement

Ethical review and approval was not required for the study on human participants in accordance with the local legislation and institutional requirements. Before the study began, written informed consent to participate in this study was provided by the regional administration of each school. These educational institutions did require written informed consent from the parents. We ensured the anonymity of the responses and the confidentiality of all data collected, with published results not containing any school identifying information.

## Author Contributions

RG designed the study, collected and analyzed the data, and wrote the manuscript. AP contributed to the interpretation of the data and wrote, revised, and refined the manuscript. RG and AP have participated in sending the article to the journal. Both authors contributed to the article and approved the submitted version.

## Conflict of Interest

The authors declare that the research was conducted in the absence of any commercial or financial relationships that could be construed as a potential conflict of interest.
